# *N*-Propargylglycine: a unique suicide inhibitor of proline dehydrogenase with anticancer activity and brain-enhancing mitohormesis properties

**DOI:** 10.1007/s00726-021-03012-9

**Published:** 2021-06-05

**Authors:** Gary K. Scott, Sophia Mahoney, Madeleine Scott, Ashley Loureiro, Alejandro Lopez-Ramirez, John J. Tanner, Lisa M. Ellerby, Christopher C. Benz

**Affiliations:** 1 Buck Institute for Research on Aging, 8001 Redwood Blvd., Novato, CA 94945, USA; 2 Department of Medicine, Center for Biomedical Informatics, Stanford University School of Medicine, Stanford, CA 94305, USA; 3 Department of Biochemistry, University of Missouri, Columbia, MO 65211, USA

**Keywords:** Proline dehydrogenase (PRODH), *N*-Propargylglycine (*N*-PPG), Anticancer drug, Brain mitohormesis

## Abstract

Proline dehydrogenase (PRODH) is a mitochondrial inner membrane flavoprotein critical for cancer cell survival under stress conditions and newly recognized as a potential target for cancer drug development. Reversible (competitive) and irreversible (suicide) inhibitors of PRODH have been shown in vivo to inhibit cancer cell growth with excellent host tolerance. Surprisingly, the PRODH suicide inhibitor *N*-propargylglycine (*N*-PPG) also induces rapid decay of PRODH with concordant upregulation of mitochondrial chaperones (HSP-60, GRP-75) and the inner membrane protease YME1L1, signifying activation of the mitochondrial unfolded protein response (UPR^mt^) independent of anticancer activity. The present study was undertaken to address two aims: (i) use PRODH overexpressing human cancer cells (ZR-75-1) to confirm the UPR^mt^ inducing properties of *N*-PPG relative to another equipotent irreversible PRODH inhibitor, thiazolidine-2-carboxylate (T2C); and (ii) employ biochemical and transcriptomic approaches to determine if orally administered *N*-PPG can penetrate the blood–brain barrier, essential for its future use as a brain cancer therapeutic, and also potentially protect normal brain tissue by inducing mitohormesis. Oral daily treatments of *N-*PPG produced a dose-dependent decline in brain mitochondrial PRODH protein without detectable impairment in mouse health; furthermore, mice repeatedly dosed with 50 mg/kg *N-*PPG showed increased brain expression of the mitohormesis associated protease, YME1L1. Whole brain transcriptome (RNAseq) analyses of these mice revealed significant gene set enrichment in *N*-PPG stimulated neural processes (FDR *p* < 0.05). Given this in vivo evidence of brain bioavailability and neural mitohormesis induction, *N*-PPG appears to be unique among anticancer agents and should be evaluated for repurposing as a pharmaceutical capable of mitigating the proteotoxic mechanisms driving neurodegenerative disorders.

## Introduction

Virtually all organisms catabolize proline using a unique and structurally conserved flavoprotein, proline dehydrogenase (PRODH); and in eukaryotes, this enzyme is associated with the inner mitochondrial membrane where it transfers two electrons to the electron transport chain to produce either ATP or reactive oxygen species (ROS) (Donald et al. 2001; Lee et al. 2003; Servet et al. 2012). PRODH’s potential importance in cancer was unknowingly revealed by Vogelstein’s group when they identified PIG6 (“p53-induced gene 6”) as one of the most strongly upregulated genes by the tumor suppressing protein, p53 (Polyak et al. 1997). Subsequently identified as mitochondrial PRODH, its critical role in maintaining tumor cell survival and anaplerotic ATP production under such microenvironmental stress conditions as hypoxia and nutrient deprivation was later demonstrated in a series of studies by Phang’s group (Pandhare et al. 2009; Liu et al. 2012; Liu and Phang 2012; Phang et al. 2012) and others (Olivares et al. 2017). Our comparison of insect and human cancer cell proline oxidation confirmed that while PRODH can induce mitochondrial ROS production, it does so by respiratory chain stimulation and not as a direct product of inner membrane PRODH enzymatic activity (Goncalves et al. 2014), supporting the concept that cancer cell context likely determines PRODH’s pivotal role as either a ROS-inducing tumor suppressor or an ATP-generating growth and survival mechanism. Today, recognizing that a key hallmark feature of cancer is its general reprogramming of normal cell metabolism (Hanahan and Weinberg 2011), investigators also appreciate that the cancer regulating role of mitochondrial PRODH must be understood within the full proline cycle, wherein the balance between proline biosynthesis (from glutamate and ornithine via P5C reduction by PYCR1) and proline catabolism (via PRODH) differentially determines cancer cell growth, death, or senescence (Phang et al. 2012; Tanner et al. 2018).

After more than a decade of global interest in cancer cell metabolism and clinical development of small molecule metabolic and mitochondrial inhibitors as a novel class of anticancer drugs (Martinez-Outschoorn et al. 2017), pharmaceutical targeting of mitochondrial PRODH has only recently come into oncologic focus (Elia et al. 2017; Scott et al. 2019). This belated interest in targeting PRODH is no doubt due to the ongoing lack of an experimentally determined human PRODH structure and an efficient means of generating recombinant human PRODH protein (Tallarita et al. 2012; Tanner 2019). Outside of oncology, the quest for PRODH inhibitors goes back more than 40 years when this was considered a possible approach to eradicate tsetse flies and prevent African trypanosomiasis (Hargrove 1976). After the first mechanism-based PRODH inhibitor was proposed in 1993 (Tritsch et al. 1993), additional progress in this area awaited report by Tanner’s group of the first crystal structure of a prokaryotic bifunctional precursor of PRODH known as PutA (Lee et al. 2003), followed by the first crystal structures of bacterial PRODH complexed with the competitive inhibitor, l-tetrahydrofuroic acid (L-THFA) (Zhang et al. 2004). Not until 14 years later did the first publication emerge showing potential anticancer activity by this reversible PRODH inhibitor (Elia et al. 2017). Administering L-THFA intraperitoneally for over two weeks at doses up to 60 mg/kg into mice bearing small orthotopic implants of breast cancer cells, these investigators demonstrated its excellent host tolerance as well as its ability to reduce pulmonary metastasis formation by 50% (Elia et al. 2017). Shortly afterwards, our group reported on yet another slightly more potent competitive PRODH inhibitor, *S*-5-oxo-2-tetrahydrofurancarboxylic acid (*S*-5-oxo); and based on structural studies from Tanner’s group (White et al. 2008; Srivastava et al. 2010) we synthesized a mechanism-based covalent inactivator of PRODH, *N*-propargylglycine (*N*-PPG), demonstrating its well tolerated systemic activity upon oral administration and its anticancer activity against a variety of human breast cancer models (Scott et al. 2019).

An important additional observation from our most recent preclinical study was that while both reversible (*S*-5-oxo, L-THFA) and irreversible (*N*-PPG) type PRODH inhibitors demonstrated similar synthetic lethality with glutaminase inhibiting and p53 upregulating drugs resulting in synergistic growth inhibition of various human cancer cell line models, only *N*-PPG produced rapid decay of PRODH protein; and this PRODH decay occurred with upregulated mitochondrial chaperone protein expression (HSP-60, GRP-75) suggesting that the PRODH-bound *N*-PPG complex uniquely activated the mitochondrial unfolded protein response (UPR^mt^) in both normal host and tumor cells, independent of *N*-PPG’s anticancer activity (Scott et al. 2019).

Thus, the present study was undertaken with two goals in mind: (i) extend our earlier observations by comparing *N*-PPG with another irreversible PRODH inhibitor, thiazolidine-2-carboxylate (T2C), to confirm that *N*-PPG uniquely induces rapid mitochondrial degradation of PRODH along with upregulated expression of chaperones and the inner membrane protease, YME1L1 (Ohba et al. 2020), consistent with activation of UPR^mt^ and enhancement of mitochondrial proteostasis (mitohormesis); and (ii) explore the drug-like ability of orally administered *N*-PPG to cross the blood–brain barrier, penetrate brain tissue sufficient to inhibit PRODH for potential treatment of primary or metastatic brain tumors, and activate the UPR^mt^ to beneficially impact normal brain function by inducing neural mitohormesis.

## Materials and methods

### Cell lines and brain extracts

The ZR-75-1 human breast cancer cell line was originally obtained from American Type Culture Collection (ATCC; Rockville, MD), frozen stocks maintained in liquid nitrogen, and cultured cells replenished every 6 months from frozen cell stocks before serial passaging beyond ten generations. Routine mycoplasma checks are performed using the MycoAlert detection kit from Lonza (Basel, Switzerland). ZR-75-1 cells are grown in ATCC recommended media under 5% CO_2_ at 37 °C. Frozen lysates of mouse brain extracts were commercially obtained from Zyagen (San Diego, CA).

### Control and *N*-PPG-treated mice

The Buck Institute for Research on Aging animal facility is an AAALAC International accredited institution (Unit Number 001070), and all procedures described below were approved by the Institutional Animal and Use Committee. Commercially obtained (BOC Sciences, Shirley, NY; batch BS16LM04253) *N-*PPG (150 mg) was dissolved in 1 mL of 0.9% saline and the solution neutralized to a pH of 6.9 with 1 M sodium hydroxide to achieve final solution volumes of *N*-PPG. We used four doses of *N*-PPG for our in vivo studies: 50, 100, 150 and 200 mg/kg administered daily to each mouse by oral gavage. Healthy appearing 5-week old male and female mice (*n* = 11) from the same mixed B6/CBA background mouse colony were randomly selected for 9 days of either *N*-PPG (*n* = 7) or saline (*n* = 4) treatments, given by oral gavage (200 μL volume) performed daily at 10am. Three mice each received repeated dosing with 100, 150, or 200 mg/kg *N-*PPG, four other mice each received daily administration of 50 mg/kg *N*-PPG, and 4 control mice each received daily administration of 200 μL saline vehicle. All mice were weighed every other day and all drug doses adjusted according to mouse weight. Between 1 and 4 h after final oral gavage on day 9, mice were anesthetized with isofluorane (Butler Schein) and cervically dislocated. Mouse brains were excised and snap frozen in liquid nitrogen immediately upon sacrifice. Total RNA for RT-PCR and RNA sequencing was triazol extracted from pulverized frozen tissue samples, as previously described (Scott et al. 2016). Total protein from the same pulverized frozen tissue samples was extracted separately, as previously reported and detailed below (Scott et al. 2019).

### Drugs, antibodies, and structural models of PRODH-drug interactions

*N*-Propargylglycine (*N*-PPG) was purchased from BOC Sciences (Shirley, NY) and thiazolidine-2-carboxylate (T2C) was purchased from Sigma-Aldrich (St. Louis, MO).

Antibodies used in this study included mouse monoclonals against β-actin (C4), PRODH (A-11), TOM20, and Rieske FeS IgG (A-5) from Santa Cruz Biotechnology (Santa Cruz, CA); HRP-conjugated goat anti-mouse secondary from Bio-Rad Laboratories, Inc. (Hercules, CA); α-tubulin mouse monoclonal (T9026) from MilliporeSigma (St. Louis, MO); YME1L1, GRP-75, and HSP-60 rabbit polyclonals from ProteinTech^™^ (Rosemont, IL); Alexa 488 goat anti-mouse and 594 goat anti-rabbit secondaries from Life Technologies (Thermo Fisher Scientific, Carlsbad, CA).

### Extraction of human cells, mitochondria, and mouse brains; and protein immunoblotting

Snap frozen organs and mouse brains were first pulverized under liquid nitrogen and then sonicated in low salt buffer supplemented with detergent (10 mM Tris pH 7.5, 50 mM NaCl, 1% SDS). Cells harvested at ~ 70% confluency were washed with ice-cold Dulbecco’s phosphate-buffered saline (DPBS) and then harvested in modified RIPA buffer (50 mM Tris–HCl (pH 8.0), 150 mM NaCl, 1% triton X-100, 0.5% sodium deoxycholate, 0.1% SDS). Lysates were sonicated then spun at 14,000 rpm for 5 min and supernatants collected. Intact mitochondria were isolated separately from freshly harvested ZR-75-1 cell cultures (two to four 145 cm^2^ dishes at ~ 90% confluence) as previously described (Goncalves et al. 2014; Scott et al. 2019); after centrifugation (10,000*g* × 10 min) the mitochondrial pellets were resuspended in STE (250 mM sucrose, 5 mM Tris–HCl, 2 mM EGTA, pH 7.4) on ice, for either PRODH enzymatic assay or immunoblotting. Prior to immunblotting, protein content was determined by Bradford Coomassie Assay (BCA) kit (Pierce, Rockford, IL) and then diluted into 2X Laemmli sample buffer. Immunoblotting was performed as previously described (Scott et al. 2019) using polyvinylidene fluoride (PVDF) membranes blocked with 5% non-fat milk in TBST (tris-buffered saline with 0.1% tween-20) incubated with primary and then secondary antibodies conjugated to horse radish peroxidase; the resulting immunoblot signals were scanned for densitometry.

### PRODH enzymatic assay

PRODH enzymatic activity was assessed on isolated ZR-75-1 mitochondria as we have previously described (Scott et al. 2019) using time-dependent fluorescence spectrometry monitoring of mitochondrial NADH levels as a function of substrate addition and after either in vivo or ex vivo mitochondrial treatment with PRODH inhibitor (*N*-PPG or T2C). This assay allows for the time-dependent generation of mitochondrial NADH immediately upon addition of proline, pyruvate or malate relative to control (no added substrate) or inhibitor. By comparing proline’s NADH generating capacity with that of another substrate such as pyruvate or malate, this assay enables the indirect bioassay of PRODH specific inhibitors relative to those also affecting other FAD-containing oxidases. To measure mitochondrial NADH formation, freshly isolated ZR-75-1 mitochondria (0.15 mg resuspended in 30 μL) were added to 165 μL of KHE buffer (120 mM KCl, 3 mM HEPES, 5 mM KH_2_PO_4_ pH 7.2) supplemented with 10 μM rotenone for a final volume of 195 μl. Replicate mitochondrial preparations were dispensed into a 96-well plate where 5 μl of vehicle or enzyme inhibitor (5 mM final concentration) were mixed into designated wells with NADH fluorescence subsequently monitored (PHERAStar FS fluorescent microplate reader; BMG LABTECH GmbH, Offenburg, Germany) with *λ*_excitation_ = 340 nm and *λ*_emission_ = 460 nm for approximately 8–10 min. Following the incubation with inhibitors, all wells were treated with 5 μL of 40 mM proline (1 mM final concentration) with NADH fluorescence followed for another 6–8 min at which point 5 μl of 40 mM malate (1 mM final concentration) was added to all wells with NADH fluorescence followed for another 6–8 min. For experiments using mitochondria from ZR-75-1 cells treated in culture with inhibitors for 48 h, measurement of NADH fluorescence was identical to above except no further addition of inhibitors.

### Laser confocal imaging

Immunofluorescent imaging of ZR-75-1 cells was performed as previously described with cells plated and then treated on 4-well glass slides (Lab-Tek®II, MilliporeSigma) using a Zeiss LSM 780 confocal microscope (Zeiss, Dublin, CA) equipped with constant temperature/CO2 regulated enclosure, under 63X oil immersion (Scott et al. 2019). Plated and treated cells were crosslinked (4% paraformaldehyde) and blocked (10% IGEPAL® CA-630 NP 40 in PBS/DEPC-water with 5% goat serum) and then probed with the indicated primary (overnight) and secondary (90 min) goat anti-mouse or anti-rabbit antibodies and then counterstained with ProLong® Gold antifade reagent with DAPI (Molecular Probes by Life Technologies, Thermo Fisher Scientific) prior to imaging.

### Semiquantitative reverse-transcription PCR (RTqPCR).

As previously described (Scott et al. 2016), total RNA was harvested using Trizol followed by treatment with DNA-free (Ambion, Austin, TX) according to the manufacturer’s specifications to remove potentially contaminating DNA. Reversed transcription was performed using 0.5 μg RNA per sample condition with Random Hexamer Primers and SuperScript II (Invitrogen, Carlsbad, CA), according to manufacturer’s specifications. PCR reactions used 1 μl aliquots from the RT reactions with Pfu polymerase (New England Biolabs, Ipswich, MA). Reaction conditions consisted of annealing at 65 °C for 10 s, extension at 72 °C for 15 s and denaturation at 96 °C for 10 s with identically prepared reactions subjected to 24–28 PCR cycles. PCR products were electrophoresed on 8% polyacrylamide gels, stained with eithidum bromide, photographed and quantified by densitometry using a GS-710 Calibrated Imaging Densitometer (Bio-Rad, Hercules, CA). Mouse specific forward and reverse PCR primers used were (5’–3’):

YME1L1 forward: AGG GAC CTT GGA TTA TCT GAACT; reverse: TGG GAT GTA TGC CAA TGG GAA.
HSP-60 forward: GCT GTA GCT GTT ACA ATG GGG; reverse: TGA CTT TGC AAC AGT GAC CC.
GAPDH forward: TGT GTC CGT CGT GGA TCT GA; reverse: CCT GCT TCA CCA CCT TCT TGAT.
β2-microglobulin forward: ATG GGA AGC CGA ACA TAC TG; reverse: CAG TCT CAG TGG GGG TGA AT.

Primer pair sequences used for confirmatory RTqPCR of neural genes (Htr3a, Itpr1, Syt6, Pcsk2) found differentially expressed by RNAseq analysis between control and treated brain samples are indicated in the legend to Supplementary Fig. S1.

### Transcriptome analysis of control and *N*-PPG-treated mouse brains

Full transcriptome RNA sequencing (RNAseq) was performed by GeneWiz (South Plainfield, NJ) using 12 μg of trizol-extracted total RNA from each of four wildtype control (B2766, B2768, B2775, B2776) and seven *N*-PPG-treated (B2761, B2762, B2764, B2770, B2772, B2773, B2774) mouse brain samples, reporting back both raw and normalized log2-scaled gene expression values (transcripts per million). Four additional aliquots of these whole brain RNA samples (two controls, two treated) were sent to GeneWiz > 8 weeks apart to serve as technical replicates for the RNAseq assessment of gene expression differences. The Genewiz bioinformatics workflow used Trimmomatic (v.0.36) to remove poor quality sequences (Bolger et al. 2014); the trimmed reads were then aligned to the *Mus musculus* GRCm38 (mm10) reference genome using STAR (v.2.5.2b) (Dobin et al. 2013). The resulting BAM files were input into featureCounts from Subread (v.1.5.2) to produce the final gene count matrix (Liao et al. 2013); and DESeq2 (v. 1.26.0) was used to normalize gene counts and perform all differential expression analyses (Love et al. 2014). Differentially expressed genes were defined as those with a rounded *p* value less than or equal to 0.05.

### Statistics

Technical and biological replicates were performed as indicated in the figure legends, and graphical plots show single, median or mean values (± SD). All replicate measures were statistically compared by one-way ANOVA F test (bar graphs), Wilcoxon analysis (violin plots), or predicted linear regressions (± 95% confidence intervals) with Pearson correlation coefficients (*R*_p_). All statistical differences were considered significant if *p* ≤ 0.05. A hypergeometric distribution (Fisher’s exact test) was used to calculate the over-representation significance of differentially expressed genes in *M. musculus* Gene Ontology (GO) pathways. Pathways with a false discovery rate (FDR)-adjusted *p* value ≤ 0.05 were considered significant. GO pathways were downloaded from the Mouse Genome Informatics repository (Law and Shaw 2018). The package ComplexHeatmap (v.2.2.0) was used to visualize gene expression (Gu et al. 2016). Reactome pathway over-representation analysis was performed by inputing the significant differentially up- and down-regulated genes (Supplementary Table 1) into the public Reactome web browser package (version 73) (https://reactome.org/PathwayBrowser/). Downstream analyses were performed using Bioconductor R (www.bioconductor.org; v.3.6.3).

## Results

### Inhibition of mitochondrial PRODH by *N*-PPG and T2C

**Fig. 1:**
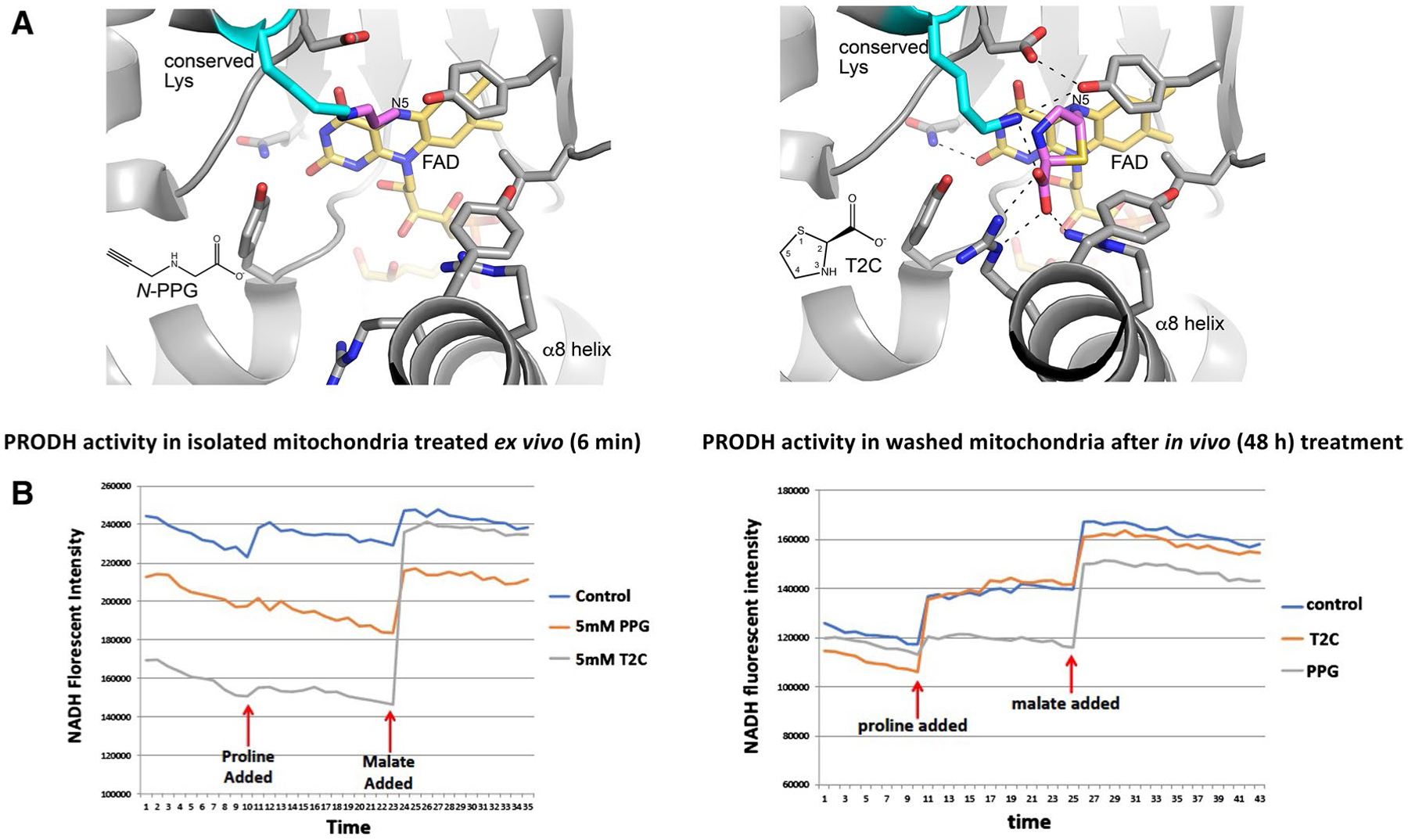
Structural models and enzymatic assays of PRODH inhibition by either *N*-PPG or T2C. **A** Structural basis for inactivation of PRODH by *N*-PPG (left panel) and the proline analog T2C (right panel). The left panel shows a crystal structure of a bacterial PRODH inactivated by *N*-PPG (Protein Data Bank ID 4NME). All the side chains shown are identically present in human PRODH. The conserved lysine (Lys234 in human PRODH) is colored cyan, the FAD is colored gold, and the 3-carbon covalent link is pink. The right panel shows the crystal structure of a bacterial PRODH inactivated by T2C (Protein Data Bank ID 6VZ9). All the side chains shown are identically present in human PRODH. The conserved lysine (Lys234 in human PRODH) is colored cyan, the FAD is colored gold, and covalently bound T2C is pink. **B** Treating isolated ZR-75-1 mitochondria with either *N*-PPG or T2C inhibits proline oxidation (left panel; *x*-axis units: 0.48 min/tick, total time shown = 17 min). However, isolating and then washing (15 min) mitochondria from control, *N-*PPG, or T2C pretreated (5 mM × 48 h) ZR-75-1 cell cultures and then assaying for NADH formation in the presence of rotenone by sequential addition of proline (1 mM) followed by malate (1 mM) shows full restoration of PRODH activity to control levels in T2C treated cells but persistent inhibition of proline oxidation and NADH formation in *N*-PPG pretreated cells (right panel; *x*-axis units: 0.48 min/tick, total time shown = 21 min)

The PRODH inhibitors *N*-PPG and T2C were used in this study. Both compounds are irreversible inactivators that covalently modify the N5 of the FAD cofactor of PRODH; however, their molecular mechanisms of inactivation differ. Inactivation by *N*-PPG results in the a 3-carbon covalent link between a conserved active site lysine (Lys234 in human PRODH) and the N5 atom of the reduced FAD cofactor (Fig. 1A, left panel). The inactivation results in stabilization of a highly open active site due to the recoil of helix α8 away from the isoalloxazine of the FAD. This mechanism of inactivation has been confirmed with several diverse bacterial PRODHs, whose pairwise sequence identity is 27–45% (White et al. 2008; Srivastava et al. 2010; Singh et al. 2014; Korasick et al. 2017). Inactivation by *N*-PPG is relatively rapid, occurring on a timescale of minutes (White et al. 2008; Srivastava et al. 2010). *N*-PPG is likely a global inactivator of PRODHs due to the high sequence conservation of the PRODH active site across both bacteria and mammals, as described previously (Tanner et al. 2018). Inactivation of PRODH by T2C is much slower (timescale of days) and results in a covalent link between the C5 atom of the T2C ring and the N5 of the reduced FAD (Fig. 1A, right panel) (Campbell et al. 2020). Unlike the *N*-PPG inactivated enzyme, the active site is closed and strongly resembles the Michaelis complex with the substrate proline, due to the structural similarity between T2C and proline. Thus, T2C-inactivated PRODH resembles a conformation of the enzyme that is sampled during the normal catalytic cycle, whereas *N*-PPG-inactivated PRODH exhibits a more distorted conformation due to the unique covalent bond between the active site lysine and the FAD. This structurally distorted PRODH-*N*-PPG mitochondrial complex is predicted to be the trigger for UPR^mt^ activation by *N*-PPG treatment of cultured cells as well as intact and healthy mammals.

We have previously described a sensitive albeit indirect measure of mitochondrial PRODH activity using isolated cancer cell mitochondria exposed ex vivo or in vivo to doses of either a reversible (L-THFA or *S*-5-oxo) or irreversible (*N*-PPG) PRODH inhibitor (Scott et al. 2019). This assay monitors the time-dependent accumulation of NADH upon mitochondrial exposure to proline (± inhibitor) in the presence of rotenone to prevent complex 1 reoxidation of NADH; and by substituting with an additional substrate this assay can also detect and compare the oxidative formation of NADH by different mitochondrial enzymes (e.g. malate dehydrogenase) confirming the specificity of PRODH inhibition. Since the proline analog thiazolidine-2-carboxylate (T2C) was recently shown in solution to be a mechanism-based inactivator of a bacterial surrogate for human PRODH (PutA), producing covalent adduction to its internal FAD (Campbell et al. 2020), we were most interested in comparing the inhibitory properties of T2C with *N*-PPG on human PRODH in isolated mitochondria from the human breast cancer cell line, ZR-75-1. Figure 1B (left panel) shows that with brief (6 min) ex vivo exposure of isolated mitochondria to a 5 mM dose of either *N*-PPG or T2C, both proline analogs produce near complete inhibition of mitochondrial proline oxidation and NADH formation, without significantly impacting malate oxidation and NADH formation. However, when looking for evidence of irreversible PRODH inhibition by pretreating cultured ZR-75-1 cells for 48 h with either *N*-PPG or T2C prior to mitochondrial isolation, Fig. 1B (right panel) shows that the isolated and washed mitochondria of in vivo T2C treated cells oxidize proline and generate NADH comparable to the mitochondria of untreated (control) cells, whereas the mitochondria from *N*-PPG-treated cells continue to show near complete and specific inhibition of proline oxidation, consistent with our previous description of *N*-PPG as an irreversible (suicide) inhibitor (Scott et al. 2019).

### *N*-PPG, but not T2C, activates mitochondrial PRODH decay, upregulates expression of chaperone proteins and the mitochondrial protease YME1L1, inducing UPR^mt^ in cancer cells

**Fig. 2:**
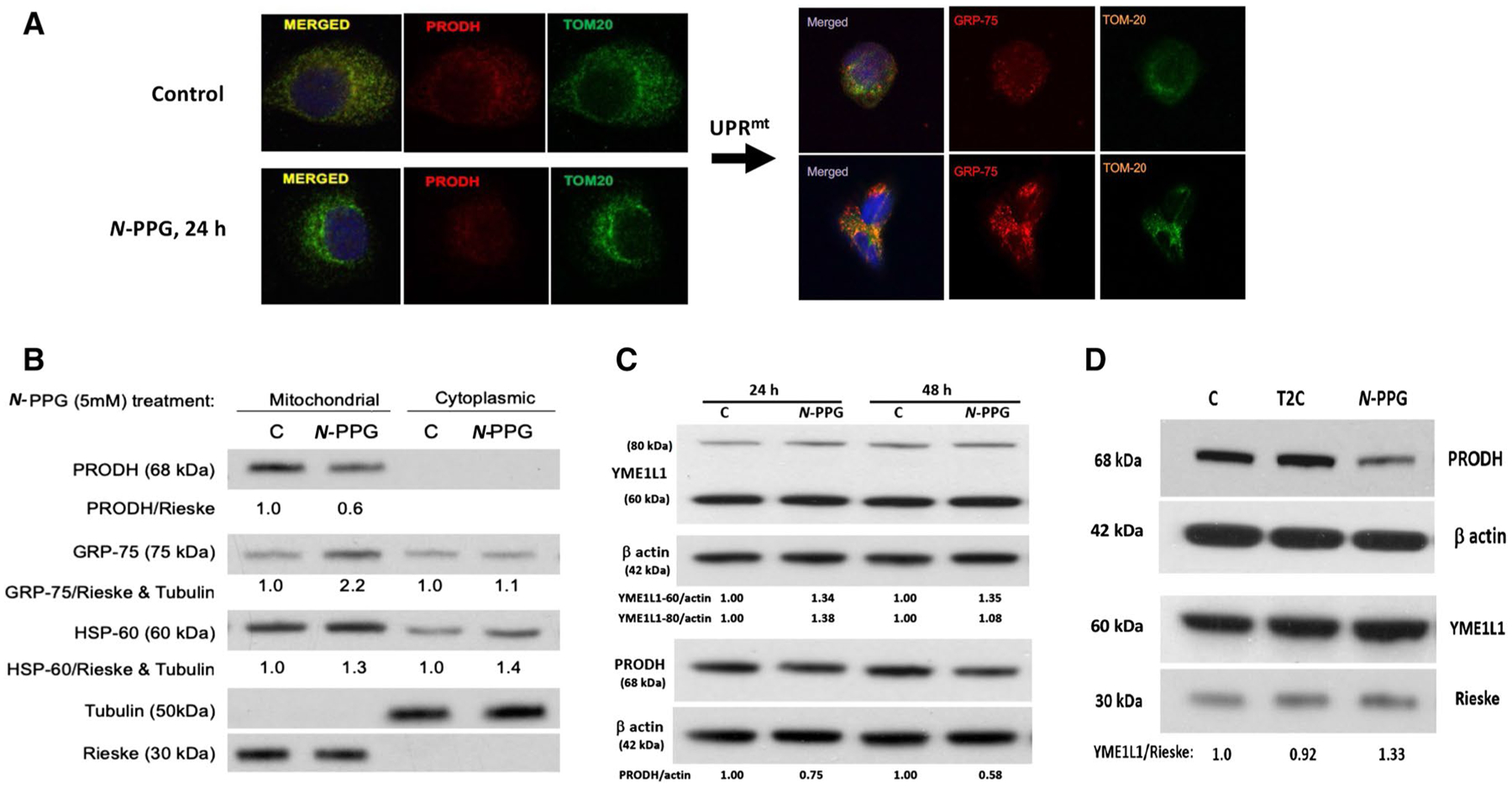
Treatment of human breast cancer cells ZR-75-1 with *N*-PPG, but not with T2C, activates mitochondrial PRODH degradation and increases mitochondrial expression of chaperone proteins (GRP-75, HSP-60) and the inner mitochondrial protease, YME1L1, consistent with UPR^mt^ induction. **A** Confocal imaging of ZR-75-1 cells (63X oil immersion magnification) treated with vehicle (control) or *N-*PPG (5 mM × 24 h), PRODH (red, left panel) or GRP-75 (red, right panel), or TOM20 (green, left and right panels) stained with mouse primaries and detected by fluorochrome-conjugated secondary antibodies. Merged images confirm mitochondrial co-localization. **B** Western blot of mitochondrial vs. cytoplasmic expression of PRODH, GRP-75, HSP-60, tubulin, and Rieske proteins 24 h after cell culture treatment of ZR-75-1 cells with *N*-PPG (5 mM) or drug vehicle (C). **C.** Western blots comparing PRODH downregulation with 60 kDa YME1L1 upregulation in whole cell lysates of ZR-75-1 cells after 24 h and 48 h exposures to *N*-PPG (5 mM) or vehicle (C). Upon import into mitochondria, cleavage of the mitochondrial targeting sequence from newly induced 80 kDa cytosolic YME1L1 produces the mitochondrial membrane-localizing and longer lasting 60 kDa YME1L1 (Hartmann 2016). **D** Western blots of ZR-75-1 whole cell lysates comparing expression of PRODH (68 kDa), YME1L1 (60 kDa), β-actin (42 kDa), and mitochondrial Rieske (30 kDa) after 24 h cell culture treatment with vehicle (C), *N*-PPG (5 mM), or T2C (5 mM)

Confocal imaging of PRODH expression in control and treated ZR-75-1 cancer cells shows not only the exclusive mitochondrial localization of PRODH protein (with TOM20 co-localization) in control cells but also its significant loss associated with upregulation of mitochondrial GRP-75 and no change in total TOM20 mitochondrial membrane expression after 24 h of 5 mM *N*-PPG treatment (Fig. 2A). In parallel immunoblotting studies, mitochondrial and cytoplasmic cell fractionation (Fig. 2B), as well as whole cell extraction (Fig. 2C, D) of ZR-75-1 cells after 24 h of *N*-PPG (5 mM) treatment, confirm the drop in mitochondrial PRODH levels concordant with upregulated expression of chaperone proteins GRP-75 and HSP-60 and both precursor (80 kDa) and mitochondrial (60 kDa) forms of the iAAA protease YME1L1 (Hartmann et al. 2016), assessed relative to tubulin, β actin, or the mitochondrial cytochrome Rieske. The observed downregulation of PRODH appears progressive when *N*-PPG culture treatment is extended from 24 to 48 h, consistent with our prior results (Scott 2019); in contrast, while mitochondrial 60 kDa YME1L1 remains constantly elevated between 24 and 48 h, its cytosolic 80 kDa precursor form is initially elevated at 24 h but then returns to near baseline levels by 48 h of *N*-PPG treatment (Fig. 2C). Consistent with our enzymatic evidence that T2C reversibly, but not irreversibly, inhibits ZR-75-1 PRODH activity (Fig. 1B), T2C appears unable to mimic the intracellular UPR^mt^ activating effect of *N*-PPG represented by mitochondrial degradation of PRODH and upregulation of 60 kDa YME1L1 (Fig. 2D).

### Oral *N*-PPG treatment of young mice reduces mouse brain PRODH protein while inducing HSP-60 and YME1L1 expression, and safely stimulates brain transcriptome neural pathways

**Fig. 3:**
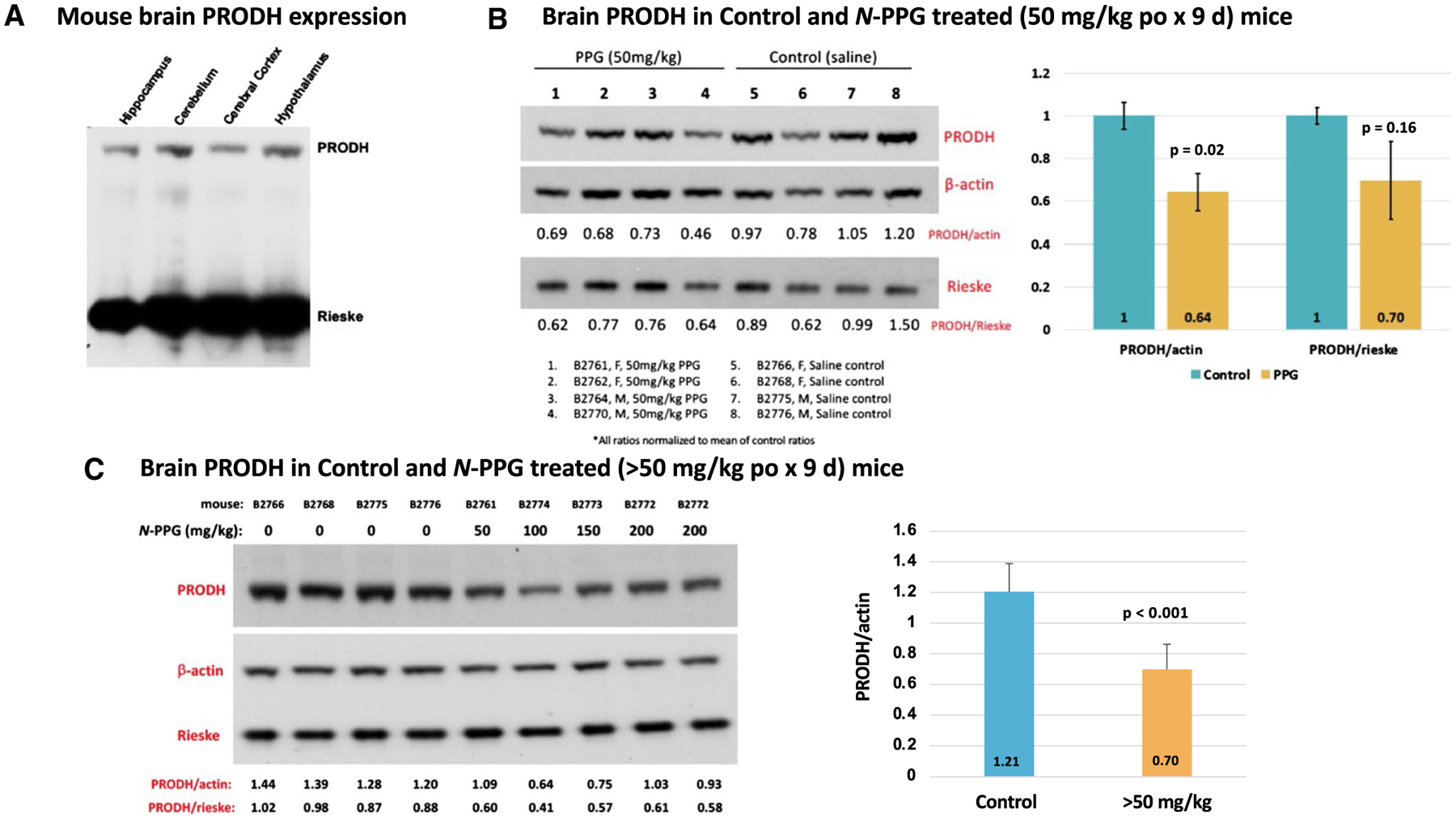
PRODH expression in mouse whole brain samples and its reduction following 9 days of oral *N*-PPG treatment. **A** Commercially obtained lysates of indicated mouse brain tissue samples immunoblotted for PRODH protein expression, with total gel protein load normalized to mitochondrial Rieske content. **B** Western blots of whole brain samples from wildtype B6/CBA mice (5 days old) orally treated for 9 days with either vehicle (saline) or 50 mg/kg *N*-PPG (left panel); image analysis quantification of probed PRODH signals normalized to either β-actin or Rieske, with bar graphs of mean ratio values (± SD) showing at least 30% reduction in mean PRODH expression following *N*-PPG treatment (right panel, ANOVA *F* test *p* values). **C.** Western blots loaded with whole brain lysates from four mice orally treated with saline (0 mg/kg *N*-PPG), one with 50 mg/kg *N*-PPG, and three with > 50 mg/kg *N*-PPG treatments (100, 150, 200 mg/kg), with an extra lane loaded with lysate from the highest dose-treated sample (left panel). Independent technical replicates of this 9-lane western blot format each probed for PRODH, β-actin and Rieske were scanned and their calculated PRODH ratios compared (right panel) to demonstrate a > 40% reduction in PRODH expression across the > 50 mg/kg treated samples relative to all saline-treated control samples (ANOVA *F* test *p* value). Western blot molecular weight standards confirmed protein running sizes as indicated in Fig. 2: PRODH at 68 kDa, β-actin at 42 kDa, and Rieske at 30 kDa

To check for brain expression of PRODH, commercially obtained mouse brain tissue lysates were immunoblotted for PRODH protein, revealing varying levels of PRODH expression in brain regions based on normalized mitochondrial Rieske loading (Fig. 3A). To explore the ability of orally administered *N*-PPG to penetrate the blood–brain barrier in sufficient amounts to induce partial degradation of brain PRODH and potentially activate the UPR^mt^ in mouse brain tissue, we chose treatment doses based on our previous mouse study wherein repeated administration of 50 mg/kg of *N*-PPG by either oral, intravenous or intraperitoneal routes was not only well tolerated but also able to downregulate PRODH protein levels in both normal host cells (kidney) and in xenografted human breast cancer cells (Scott et al. 2019). Given those earlier findings, young (5 days old) wildtype mice of both sexes were gavaged with increasing doses of *N*-PPG beginning at 50 mg/kg, or saline vehicle alone, for nine sequential days; seven mice received daily treatments with either 50 (*n* = 4), 100 (*n* = 1), 150 (*n* = 1) or 200 (*n* = 1) mg/kg *N*-PPG. Immunoblotting brain protein extracts from the four 50 mg/kg treated mice showed 64–70% of control brain PRODH expression, depending on normalization to either actin or Rieske loading (Fig. 3B). In contrast, technical immunoblot replicates quantitating PRODH/actin expression ratios from four saline-treated control mouse brains (B2766, B2768, B2775, B2776), a representative mouse brain sample receiving 50 mg/kg treatment (B2761), and three mouse brain samples receiving > 50 mg/kg treatments (B2772-200 mg/kg, B2773-150 mg/kg, and B2774-100 mg/kg) showed that mouse brains exposed to higher *N*-PPG doses (> 50 mg/kg) expressed only 58% *(p* < 0.001) of control brain PRODH levels, suggesting slightly greater reduction in brain PRODH levels following higher dose oral *N*-PPG treatments (Fig. 3C).

**Fig. 4:**
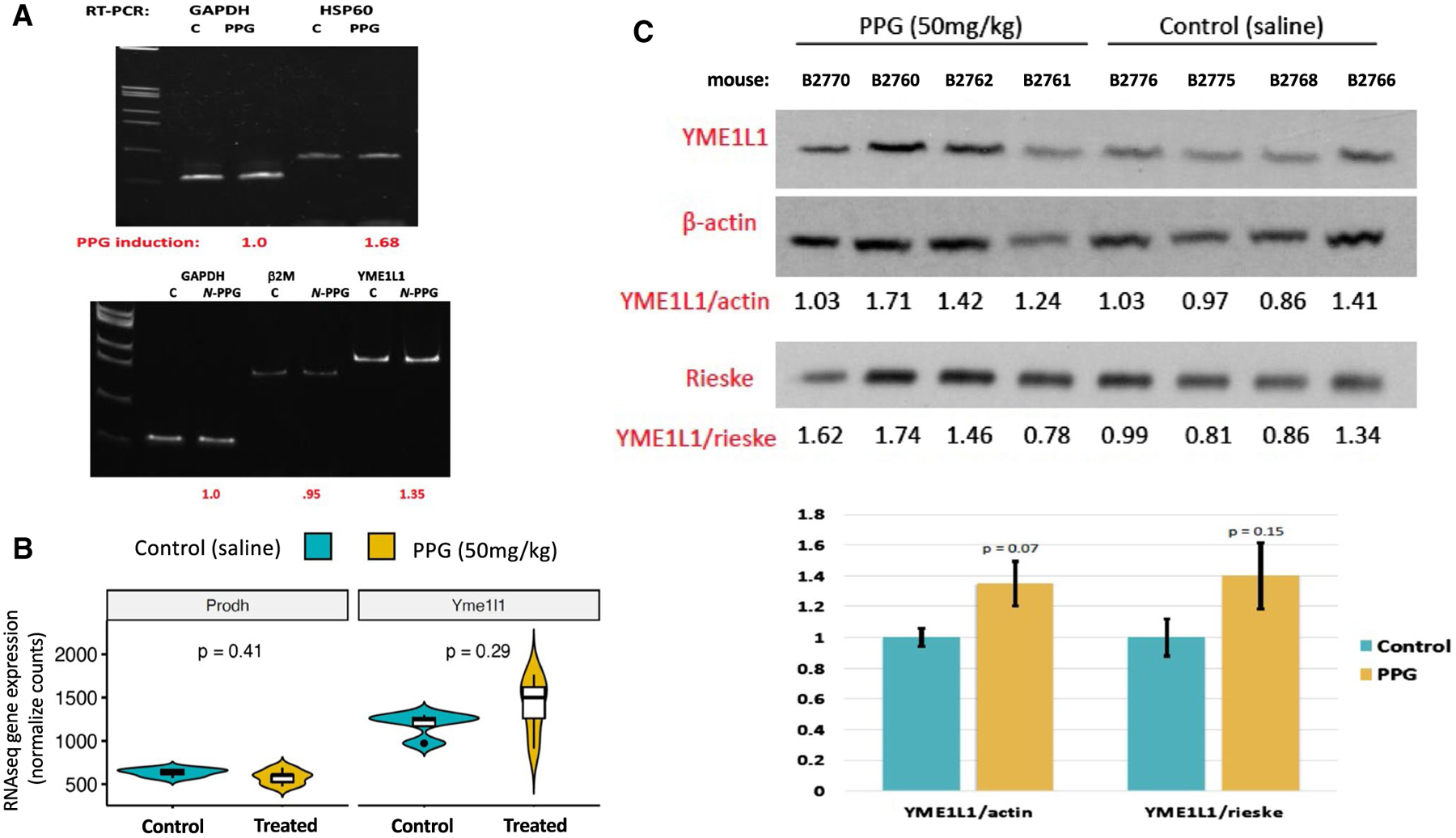
Oral *N*-PPG treatment induces expression of the mouse brain mitochondrial protease YME1L1**. A** Total RNA extracted from single control (C) and 200 mg/kg *N*-PPG-treated mouse brains analyzed by RTqPCR to show 1.7-fold induction of chaperone HSP-60 transcripts and 1.4-fold induction of mitochondrial protease YME1L1 transcripts relative to housekeeping transcripts (GAPDH and β2 M) following 9 days of oral *N*-PPG treatment. **B** Violin plots (median: dark line, open box: Q1–Q3, distribution with max./min. values) of PRODH and YME1L1 gene expression values from RNAseq analysis of brain samples from all four control and all four 50 mg/kg *N*-PPG-treated mice (p values determined by Wilcoxon test). **C** Western blots of whole brain protein lysates from all saline-treated control and 50 mg/kg *N*-PPG-treated mice, as analyzed in Fig. 3B, with bar graphs below western blots showing image analysis quantification (mean ratio values ± SD) of 80 kDa YME1L1 expression normalized to either 42 kDa β-actin or 30 kDa Rieske, indicating a near 40% increase in mean YME1L1 expression following *N*-PPG treatment (ANOVA *F* test *p* values)

Brain RNA and protein extracts were also interrogated for evidence of *N*-PPG induced UPR^mt^ beyond the observed dose-dependent decline in PRODH protein expression. RTqPCR was performed on total RNA extracted from a representative control brain sample for comparison with the 200 mg/kg treated brain sample, showing that *N*-PPG upregulated HSP-60 (1.68-fold) and YME1L1 (1.35-fold) transcript levels relative to either β2-microglobulin or GAPDH transcript levels (Fig. 4A). Next, we compared PRODH and YME1L1 mRNA and protein expression between our four control and four 50 mg/kg treated mouse brain samples. The median levels of PRODH mRNA appeared basically unchanged while median levels of YME1L1 transcripts increased nominally (but not significantly) by 18.5% following the *N*-PPG treatment (Fig. 4B), consistent with *N*-PPG induced post-transcriptional decay of mitochondrial PRODH protein associated with transcriptional induction of the YME1L1 protease. Looking at protein expression in these same brain samples, mean brain expression of YME1L1 increased nominally from 30% (normalized to actin, *p* = 0.07) to 40% (normalized to Rieske, *p* = 0.15) following the 9 days of oral 50 mg/kg *N*-PPG treatment (Fig. 4C).

**Fig. 5:**
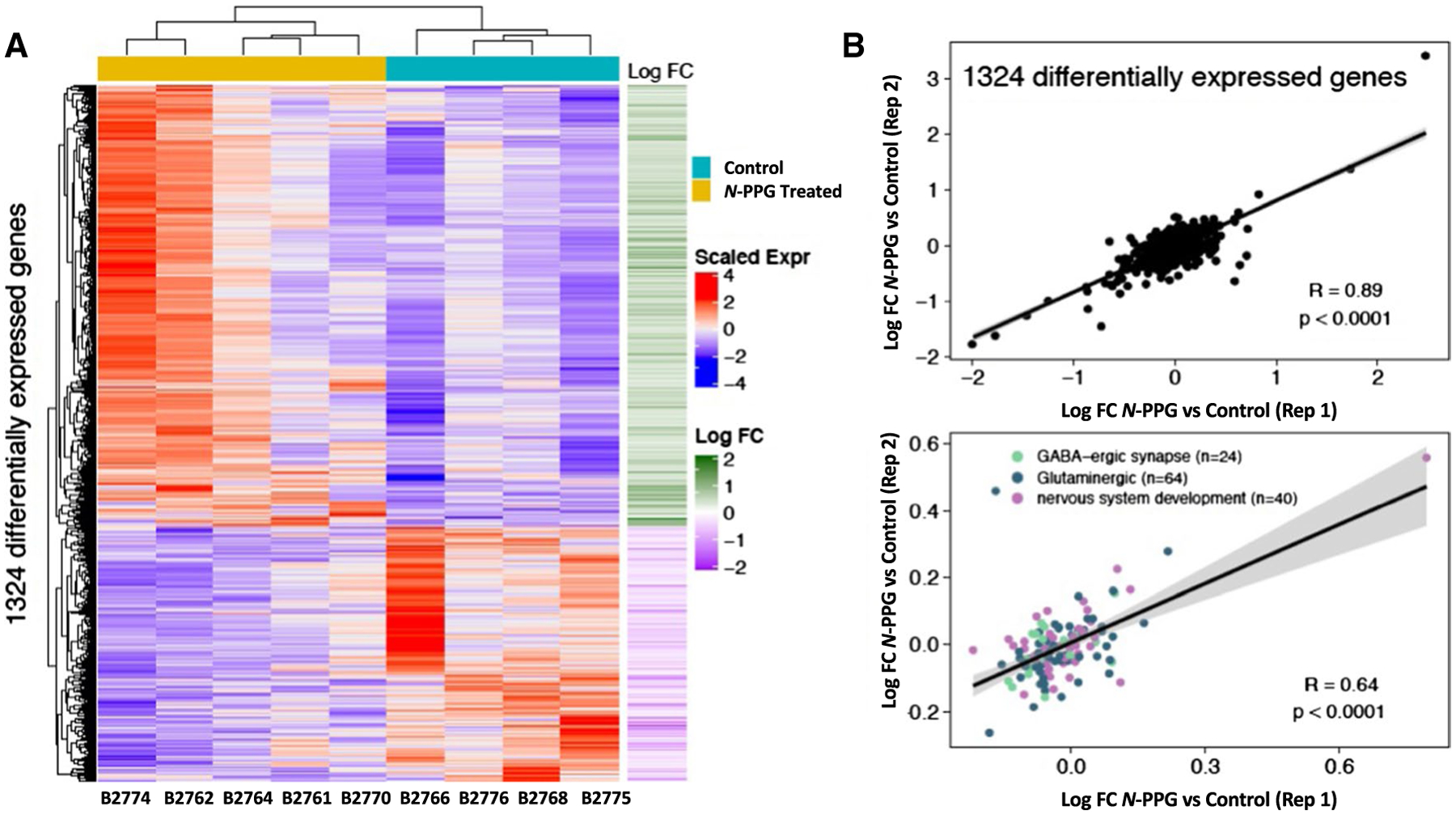
Transcriptomic comparison of control and *N*-PPG-treated mouse brains**. A** Gene expression heat map and unsupervised hierarchical clustering of 1324 genes found to be differentially expressed (*p* < 0.05; Supplementary Table 1) between five *N*-PPG-treated mouse brains and four saline-treated control brains. All treated mice received 9 days of 50 mg/kg *N*-PPG except B2774 who received 100 mg/kg *N*-PPG. Scaled gene expression is shown alongside log-fold changes (Log FC). Upregulated genes (red on heatmap and corresponding green Log FC) include 841 of the 1324 differentially expressed genes; and by over-representation analysis these encode 77 enriched Gene Ontology (GO) pathways (Supplementary Table 2) and 7 Reactome neural pathways (Supplementary Table 4) having FDR significance *p* < 0.05. **B** Pearson correlations (*R*_p_) and linear regressions of Log FC gene expression differences between blinded RNAseq analysis of treated and control technical replicates (Reps 1 and 2) as described in Methods: (top plot) across all 1324 differentially expressed genes; (bottom plot) for the 127 non-redundant over-represented genes comprising enriched GO terms for nervous system development (*n* = 40), glutaminergic (*n* = 63) and GABAergic (*n* = 24) synapses (Supplementary Table 3). Grey bands around the predicted regression lines represent 95% confidence intervals

To assess the genomic impact of *N*-PPG treatment on the entire brain transcriptome, beyond its targeted effects on mitochondrial PRODH protein and induction of UPR^mt^ components, we employed full transcriptome RNAseq to identify the subset of differentially expressed genes among all 17,173 brain genes expressed in four control and five *N*-PPG-treated mouse brains. Importantly, our RNAseq technical replicates (Rep 1 and Rep 2) showed excellent concordance between their log-fold differences across all expressed genes (*n* = 17,173 genes; *R*_p_ = 0.87, *p* < 0.0001). Unsupervised hierarchical clustering was performed on the 1324 genes differentially expressed at *p* ≤ 0.05 significance between our five *N*-PPG-treated mouse brains and our four saline-treated control brains (Fig. 5). Figure 5A shows a heat map of the 1324 differentially expressed genes and the two well-defined clusters of control and *N*-PPG-treated brain samples, along with the log-fold changes in the 841 *N*-PPG upregulated genes relative to the 483 *N*-PPG downregulated genes (identified in Supplementary Table 1). Over-representation analysis of these transcriptome differences identified 77 significant (FDR *p* < 0.05) Gene Ontology (GO) pathways represented by the 841 *N-*PPG upregulated genes and only 6 significant (FDR *p* < 0.05) GO pathways represented by the 483 N-PPG downregulated genes (Supplementary Table 2). The most significant GO upregulated pathways point almost exclusively to stimulated neural cell function, primarily glutaminergic and GABAergic synapses, receptors and signaling components, and secondarily to specific voltage-gated ion channel pathways (Supplementary Table 2). The neural pathway GO genes differentially expressed after *N*-PPG treatment are listed according to their GO group significance level as well as by their expression level in each individual mouse brain (Supplementary Table 3). Reactome analyses, by contrast, identified no significant pathway enrichment for the *N*-PPG downregulated genes, but significant enrichment (FDR, *p* < 0.05) in 7 exclusively neural pathways for the *N*-PPG upregulated genes (Supplementary Table 4). As shown in Fig. 5B, RNAseq technical replicates (Rep 1 and Rep 2) showed excellent concordance between their log-fold differences across the 1324 differentially expressed genes (*R*_p_ = 0.89, *p* < 0.0001); as well as across the 127 over-represented and non-redundant genes (*R*_p_ = 0.64, *p* < 0.0001) comprising enriched GO terms for nervous system development (*n* = 40 genes), glutaminergic (*n* = 63 genes) and GABAergic (24 genes) synapses. To spot check our RNAseq finding of *N*-PPG stimulated neural system pathways, glutaminergic and GABAergic synaptic functions, two residual control samples and two residual *N*-PPG-treated brain tissue samples were re-extracted for RNA and analyzed by RTqPCR to confirm induction of four different genes represented within the most significantly stimulated neural GO pathways (highlighted in Supplementary Table 3): Htr3a (5-Hydroxytryptamine receptor 3A), Itpr1 (Inositol 1,4,5-trisphosphate receptor type 1), Syt6 (Synaptotagmin-6), and Pcsk2 (Proprotein convertase subtilisin/kexin type 2). While our RNAseq data (normalized counts) showed a mean 1.14- to 1.32-fold induction of these four genes across the *N*-PPG-treated samples (Supplement Table 3), our spot check RTqPCR analyses showed that *N*-PPG stimulated their mean expression (normalized to GAPDH) as follows: 1.64 for Htr3, 1.72 for Itpr1, 1.48 for Syt6, and 2.00 for Pcsk2 (Supplemental Fig. S1).

## Discussion

Specific reversible (competitive) and irreversible (suicide) inhibitors of mammalian PRODH have recently been shown to inhibit cancer cell growth in vitro and in vivo, with excellent host tolerance and no systemic side effects (Elia et al. 2017; Scott et al. 2019). Independent of its anticancer activity and in all PRODH expressing cells and tissues studied to date, the orally bioavailable PRODH suicide inhibitor, *N*-propargylglycine (*N*-PPG), appears to be functionally unique based on its rapid and selective decay of mitochondrial PRODH protein along with its upregulated expression of mitochondrial chaperone proteins, HSP-60 and GRP-75, and the mitochondrial inner membrane protease, YME1L1—altogether signifying an activated mitochondrial unfolded protein response (UPR^mt^) with enhanced mitochondrial proteostasis (Jensen and Jasper 2014; Wiechmann et al. 2017; Sorrentino et al. 2017; Münch et al. 2018; MacVicar et al. 2019; Ohba et al. 2020). Despite having similar anticancer activity as *N*-PPG, the competitive PRODH inhibitors L-THFA and *S*-5-oxo both fail to induce PRODH decay and upregulate HSP-60, GRP-75, or YME1L1 (Scott et al. 2019). Thiazolidine-2-carboxylate (T2C) was recently found to irreversibly bind and inhibit PRODH upon extended exposure in solution (Campbell et al. 2020); and we now observe that when exposed to intact human mitochondria ex vivo, T2C and *N*-PPG similarly inhibit PRODH activity, but upon extended (48 h) in vivo exposure to human cells in culture only *N*-PPG exhibits the intracellular characteristics of an irreversible mechanism-based PRODH inhibitor. Previous structural modelling suggested that *N-*PPG’s unique ability to induce mitochondrial PRODH decay and activation of the UPR^mt^ is likely due to its post-reactive covalent linkage between the pocket’s FAD N5 atom and a conserved active site lysine residue, causing irreversible structural distortion to the overall PRODH-bound complex (Scott et al. 2019). This hypothesis is consistent with model organism experiments in which UPR^mt^ is often activated by introducing structural distortion in a single intramitochondrial protein (Jensen and Jasper 2014).

It is notable that T2C behaved like a reversible inhibitor in our *in cellulo* experiments, yet it is an irreversible covalent inactivator of purified bacterial PRODH. The atypical inactivation mechanism of T2C (Campbell et al. 2020), which includes competing pathways and a very long inactivation timescale, may account for these differences. The proposed mechanism of T2C involves two pathways, one beginning with the oxidation of the C4 atom of T2C, which does not lead to covalent inactivation, and another beginning with the oxidation of T2C at C5, which results in the covalent modification of the FAD shown in Fig. 1A (right panel). These two oxidation pathways result in very rapid reduction of the FAD, which implies that T2C initially behaves like a reversible competitive inhibitor by occupying the proline-binding site. It is possible that the apparent inhibition observed in mitochondria treated ex vivo reflects this reversible binding of T2C to PRODH (Fig. 1B, left panel). Another unusual feature of the T2C inactivation mechanism is the multi-day lag between the reduction of the FAD (by the pathways described above) and the appearance of covalent inactivation. In our 48 h in vivo experiments (Fig. 1B, right panel), the oxidized T2C species would be exposed to the complex environment of the cell, and it is possible that the C5-oxidized T2C is eliminated by cellular processes before covalent inactivation can occur. In contrast, with *N-*PPG the timescales for FAD reduction and inactivation are commensurate, occuring within minutes, so its reactive intermediates may be less vulnerable to cellular processes.

While there are many mitochondrially targeted drugs under clinical development, few have become clinically approved anticancer agents (Martinez-Outschoorn et al. 2017; Chiu et al. 2020; Jeena et al. 2020), none capable of direct UPR^mt^ induction (O’Malley et al. 2020) and few showing complete absence of systemic host toxicity no doubt because normal tissues frequently have activated metabolic pathways like cancer, making dose-limiting host toxicity a challenge when targeting those pathways (Martinez-Outschoorn et al. 2017). Relative to the UPR^mt^ pathway, targeting the mitochondrial chaperone HSP-60 has recently been shown to be a promising new anticancer strategy with low toxic impact on normal cells (Wiechmann et al. 2017); but this antimitochondrial agent would be expected to blunt if not prevent UPR^mt^ activation. Rather, induction of UPR^mt^ has been proposed as a possible new cancer treatment strategy, albeit in need of selective and direct acting inducers (Siegelin et al. 2011; O’Malley et al. 2020). In this regard, *N*-PPG appears to be both an anticancer agent and a direct acting UPR^mt^ inducer, given that inhibition of PRODH function is deleterious to malignant but not normal mammalian cells (Elia et al. 2017; Scott et al. 2019).

We have previously shown that *N*-PPG is systemically well distributed when administered by oral, intraperitoneal or intravenous routes (Scott et al. 2019). Following our earlier observed absence of any systemic toxicity despite repeated in vivo administration of *N-*PPG sufficient to induce UPR^mt^ in both normal host and xenografted malignant cells, we wondered if its UPR^mt^ inducing effect on normal host cells might in fact be systemically beneficial given growing recognition that enhancing mitochondrial proteostasis (mitohormesis) in normal cells is often beneficial to normal organismal growth and development, improving cell vitality and even extending lifespan (Jensen and Jasper 2014; Moehle et al. 2019). Thus, the present mouse study was also undertaken to further explore the bioavailability potential of systemically administered *N*-PPG as well as its ability to induce UPR^mt^ and mitohormesis in a sanctuary organ site like the brain, normally protected from most chemotherapeutic agents by the blood–brain barrier.

Having demonstrated the strict target selectivity of *N*-PPG for PRODH by showing its inability to inhibit other mitochondrial or cytosolic flavoproteins (Scott et al. 2019), we were encouraged to look into its brain penetrating potential since another propargylic (*N*-PPG-like) analog, rasagiline, a clinically approved selective and irreversible inhibitor of the FAD-containing monoamine oxidase (MAO-B), readily crosses the blood–brain barrier and is used to treat Parkinson’s disease (Binda et al. 2005). Indeed, our current 9 day trial of sequential oral treatment of mice with 50 mg/kg *N-*PPG proved sufficient to diminish overall PRODH levels in the mouse brain by at least 30% (Fig. 3B); and additional experiments suggested that dosing mice with > 50 mg/kg *N*-PPG (100–200 mg/kg) might further reduce brain PRODH levels by more than 40% without producing any overt systemic or neurologic host toxicity (Fig. 3C). Since brain metastases represent a common lethal outcome for patients with melanoma, lung and breast cancers (Preusser et al. 2012; Dagogo-Jack et al. 2016), and since PRODH overexpression is associated with the most aggressive and life-threatening form of primary brain glioblastomas (Panosyan et al. 2017), the promising ability of *N*-PPG to cross the blood–brain barrier and downregulate PRODH expression deserves further study in preclinical models of primary and metastatic brain tumors.

As expected, the *N*-PPG induced decline in overall brain PRODH protein expression appeared to be accompanied by upregulated mitochondrial chaperones like HSP-60 and the inner mitochondrial membrane protease, YME1L1, whose evolutionarily conserved function is to not only protect mitochondrial integrity by degrading its structurally damaged proteins but also to mediate UPR^mt^ (Sorrentino et al. 2017) and enhance mitochondrial plasticity and proteostasis (MacVicar et al. 2019; Ohba et al. 2020), the loss of which is a common pathogenic feature driving many proteotoxic neurodegenerative diseases (Sorrentino et al. 2017; Lautenschlager 2020). Consistent with our previous results (Scott et al. 2019), this study’s 9 day course of oral *N*-PPG treatment proved to be entirely free of any adverse systemic or neurologic consequences, likely due to its partial rather than complete knockdown of brain PRODH levels and despite the fact that our full brain transcriptome analysis confirmed the significant global stimulatory impact of *N*-PPG treatment on such key neural transmission pathways as glutaminergic and GABAergic synapses (Fig. 5; Supplementary Fig. S1 and Table 3). This study (Fig. 3A) and others (Panosyan 2017) confirm that both the mouse and human brain express PRODH protein; however, the Human Brain Atlas suggests that both PRODH mRNA and protein expression exhibit significant regional and cell type specificity (https://www.proteinatlas.org/ENSG00000100033-PRODH/brain). Therefore, more detailed neuropathologic and neurofunctional mouse studies are now essential to identify specific neural cell types and brain compartments most susceptible to *N*-PPG treatment, and to explore the possibility that the brain mitohormesis inducing properties of this drug are sufficiently beneficial to warrant its repurposing from a potential anticancer agent to a much needed new treatment to prevent or mitigate the proteotoxic mechanisms driving various neurodegenerative disorders.

## Supplementary Material

Supplementary Table 1

Supplementary Table 2

Supplementary Table 3

Supplementary Table 4

Supplementary Figure S1
