# Supplementary figures and images for: N-Propargylglycine: a unique suicide inhibitor of proline dehydrogenase with anticancer activity and brain-enhancing mitohormesis properties
Source: Amino Acids. 2021 Jun 5;53(12):1927–39. doi: 10.1007/s00726-021-03012-9 (PMC8643368; doi:10.1007/s00726-021-03012-9)

### Figure S1

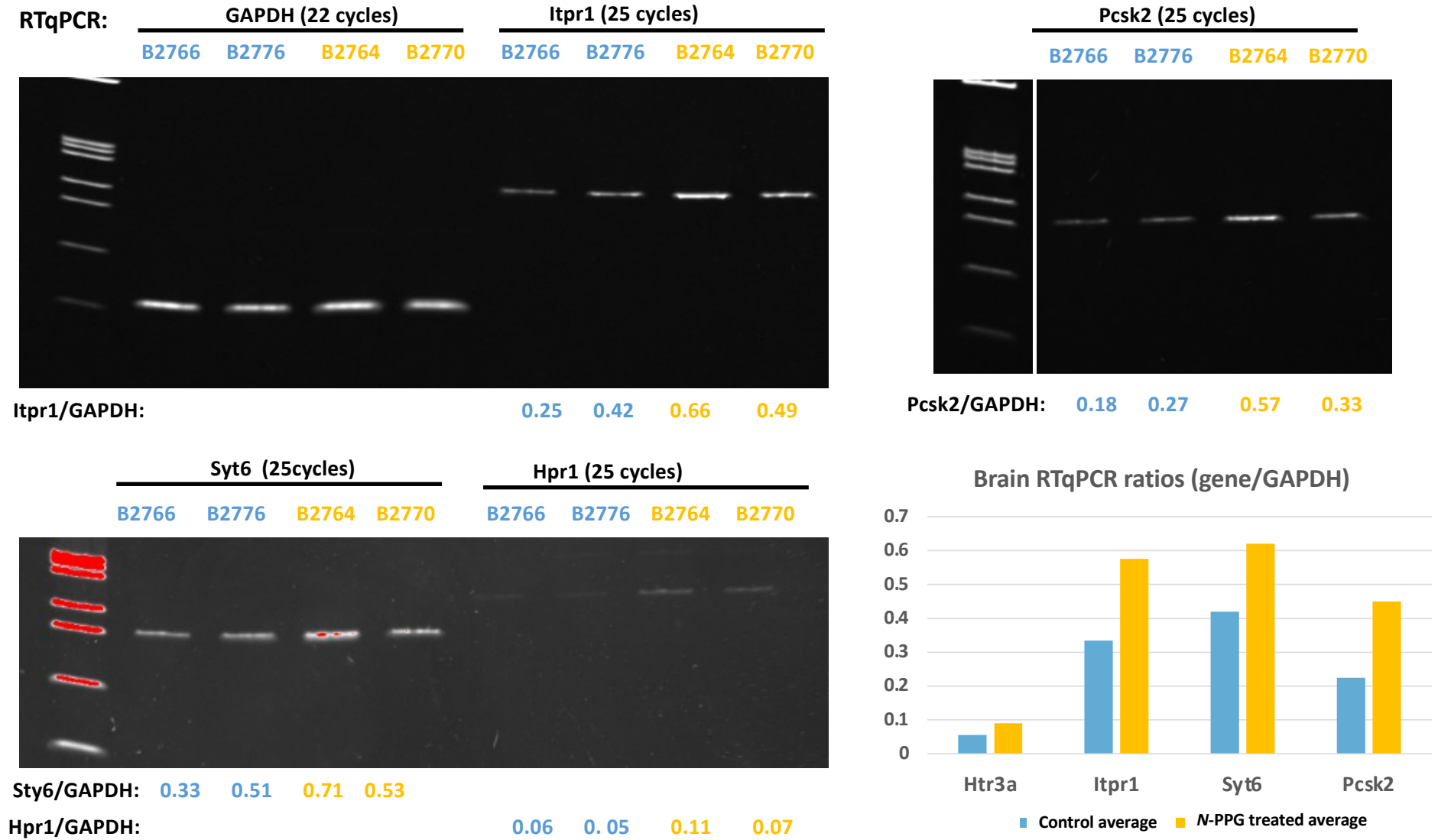

Supplement: Supplementary file 1 — Supplementary Figure S1. RTqPCR confirmation of N-PPG treatment induction of four mouse brain expressed genes (Htr3a, Itpr1, Syt6, Pcsk2) identified by RNAseq analysis and representing the most significantly enriched neural GO pathways. Total RNA extracted from two frozen control (B2766, B2776) and two frozen 50 mg/kg N-PPG treated (B2764, B2770) mouse brain tissue samples were analyzed by RTqPCR for expression of the housekeeping transcript GAPDH and four genes (Hlt3a, Itpr1, Syt6, Pcsk2) representing neural system pathways, glutaminergic and GABAergic synaptic functions as highlighted in Supplementary Table 3. Ratios of the amplified neural gene band intensities normalized to GAPDH are shown beneath each gel (blue = saline treated, yellow = N-PPG treated). When these gene expression ratios are averaged over the control and treated mouse groups (summary bar graph, lower right) they confirm that N-PPG treatment stimulated brain expression of the neural genes as follows: 1.64 for Htr3, 1.72 for Itpr1, 1.48 for Syt6, and 2.00 for Pcsk2. Mouse specific PCR primer pairs used were (5’ to 3’): Htr3 forward: GCCGGAGGCCTTTATTCTACGCAGTC ; Htr3 reverse: GAAGATGGTCTCAGCGAGGCTTATCAC. Itpr1 forward: GGAAGACAGGGACATCCTCAGCTAC; Itpr1 reverse: CTGACCACAGTCGGGCATATTTCACA . Syt6 forward: CATGTCTCCAGCGTGGACTATGGC; Syt6 reverse: GCACAATCAGCGTCTCGCTTTCAT. Pcsk2 forward: CTGTGACGGCTATGCTTCAAGCATG; Pcsk2 reverse: CTGCAGATGTCCCAGAGTGTCTCAGAG. GAPDH forward: TGTGTCCGTCGTGGATCTGA; GAPDH reverse: CCTGCTTCACCACCTTCTTGAT [file 726_2021_3012_MOESM1_ESM.pdf]
